# Implementation process evaluation and preliminary effect analysis of an outpatient multidisciplinary follow-up program for adolescents with acute alcohol intoxication in Belgium: the SPIRIT pilot study

**DOI:** 10.1186/s13722-025-00629-z

**Published:** 2025-12-11

**Authors:** Hanna van Roozendaal, Inge Glazemakers, Lieve Verboven, Brecht De Tavernier, Ann Vander Auwera, Els Verlinden, Frederic De Meulder, Marjolein Mattheij, Stijn Verhulst, Jozef De Dooy, Nico van der Lely, Guido Van Hal

**Affiliations:** 1 Faculty of Medicine and Health Sciences, University of Antwerp, Wilrijk, Antwerp, 2610 Belgium; 2 University Centre for Child and Adolescent Psychiatry (ZAS-UKJA), Antwerp, 2020 Belgium; 3 Department of Mental Health Care for Children and Adolescents, ZAS Hospitals, Wilrijk, Antwerp, 2610 Belgium; 4 Emergency Department, ZAS Hospitals, Wilrijk, Antwerp, 2610 Belgium; 5 Department of Paediatrics, ZAS Hospitals, Wilrijk, Antwerp, 2610 Belgium; 6 Department of Medical Management, ZAS Hospitals, Wilrijk, Antwerp, 2610 Belgium; 7 Department of Paediatrics, Antwerp University Hospital, Edegem, Antwerp, 2650 Belgium; 8 Department of Paediatrics, Reinier de Graaf Hospital, Delft, 2625 AD The Netherlands

**Keywords:** Alcohol drinking, Alcohol intoxication, Adolescent, Psychosocial intervention, Implementation science, Consolidated framework for implementation research, Qualitative research, Quasi-experimental study, Belgium

## Abstract

**Background:**

The number of adolescents with acute alcohol intoxication in Belgium is concerning and standardised follow-up care for these adolescents is lacking. Therefore, a six-month follow-up treatment program called SPIRIT, which stands for Screening and Personalised Feedback Intervention for Alcohol-Intoxicated Teenagers), was developed and pilot-tested in Antwerp (Belgium) in this study. The program consisted of personalised feedback, motivational interviewing, psychological screening, and parental involvement,

**Methods:**

The primary objective of this pilot study was to evaluate the implementation process of SPIRIT, guided by the Consolidated Framework for Implementation Research (CFIR). Interviews with paediatricians, brainstorming meetings with stakeholders, and participant surveys were conducted and analysed qualitatively. Thematic content deriving from the qualitative data coding was linked to CFIR domains and relevant constructs, which were assessed as factors influencing implementation, relating to (1) barriers and (2) facilitators to implementation. The study’s secondary objective consisted of the preliminary analysis of SPIRITs effectiveness in decreasing problematic alcohol use, parent-child interaction, parenting skills, and the detection of underlying psychological disorders. Surveys for participants and their parents were conducted at the moment of discharge from the emergency department (T0) and at follow-up six months later (T2).

**Results:**

The qualitative analysis revealed that 2 of the CFIR constructs were considered barriers for implementation, namely the constructs financing (outer setting) and sustainability (outcome addendum). Fifteen constructs were considered as a combination of a facilitator and barrier (mainly the domains ‘outer setting’, ‘inner setting’ and ‘individuals’). The results also showed a strong innovation design and implementation process, with 21 CFIR constructs serving as facilitators in mainly these two above-mentioned domains. Additionally, the quasi-experimental effect analysis showed a decrease in problematic alcohol use among the 12 participants (from a median AUDIT-C score of 4.0 to 1.0, *p* = 0.013). Also, an improvement in the conflict behaviour between participants (the median Conflict Behaviour Questionnaire, CBQ, score decreased from 6.0 to 1.5, *p* = 0.044) and their mothers (median CBQ decreased from 8.5 to 3.5, *p* = 0.037) was seen. Moreover, a significant increase in the parenting skills of fathers was observed (a decrease in mean Parenting Style score from 3.3 to 2.7, *p* = 0.046).

**Conclusions:**

Despite the low sample size, the preliminary effect analysis of this study demonstrated promising results in decreasing problematic alcohol use and improving parent-child interaction. The barriers (mainly in the inner and outer setting domains, for instance patient flow and financing) and facilitators (mainly in the innovation and implementation process domain) assessed in this study using the CFIR framework, could be considered by researchers, physicians and policy makers when implementing similar follow-up programs in healthcare contexts.

**Trial registration:**

ISRCTN, ISRCTN15542211, 22/04/2025, retrospectively registered.

**Supplementary Information:**

The online version contains supplementary material available at 10.1186/s13722-025-00629-z.

## Background

Alcohol misuse among adolescents is a significant public health concern [1]. Globally, alcohol use is the leading cause of death and the highest risk factor for disability−adjusted life−years among individuals aged 15–19 years [2]. In Europe, the prevalence of drinking in this age group (43.8%) is the highest in the world [1]. When adolescents drink, they often drink in heavy drinking sessions [1]. This is also seen in Belgium, where 30% of the Flemish 17–18-year-olds binge drink at least once a month [3]. Binge drinking is defined as consuming ≥6 units of alcohol in two hours for males and ≥4 units in two hours for females [3]. While the prevalence and frequency of alcohol use among Belgian adolescents are decreasing, binge drinking rates have remained unchanged [3]. This is also reflected in the concerning number of adolescents with acute alcohol intoxication (AAI). Every year, 31 per 10,000 adolescents aged 10–17 in Antwerp, Belgium’s second-largest city, are admitted to the hospital with AAI [4]. On the national level, the prevalence of AAI among adolescents has been estimated to be even higher [5]. As AAI can lead to severe complications, such as reduced consciousness, hypothermia and metabolic acidosis, medical management and monitoring are often needed [6]. Alcohol-related medical complications are more frequently present in adolescents compared to adults. [7]. Furthermore, these episodes are linked to injuries, traffic accidents and delinquent behaviours [8, 9]. Long-term risks include alcohol use disorder [10], alcohol-related cancers [11, 12], brain damage [13], mental health disorders [14], and academic decline [15].

Reducing alcohol-related harm among adolescents with problematic alcohol use requires the implementation of effective interventions [16]. Psychological interventions using motivational interviewing (MI) have proven effective in reducing alcohol consumption in adolescents with AAI [17–19]. However, no such follow-up programs exist in Belgium. This lack of evidence-based treatment options available to Belgian adolescents presenting to hospital with AAI highlights an important gap in service provision. Simply presenting to hospital following AAI does often not result in behaviour change amongst adolescents [20, 21]. Therefore, Belgian adolescents with AAI are at risk for persisting problematic alcohol-drinking behaviour and recurrent AAI admissions. Furthermore, underlying mental problems may go undetected without additional psychological screening. This is specifically relevant as earlier research showed a relatively high incidence of comorbid mental disorders among adolescents with AAI [14, 22, 23]. Finally, the absence of follow-up care misses an opportunity to motivate parents to adopt stricter attitudes and monitor their child’s drinking behaviour when needed, which are known to be protective factors against alcohol misuse [24, 25].

To address this gap, we developed and pilot-tested the first outpatient intervention for adolescents admitted with AAI to emergency departments (EDs) in Belgium. This six-month follow-up treatment program, the SPIRIT intervention (Screening and Personalised Feedback Intervention for Alcohol-Intoxicated Teenagers), combines personalised feedback, MI, psychological screening, and parental involvement. The intervention is based on the already existing and similar program ‘Jeugd en Alcohol’ in the Netherlands [22, 26], but adjusted to the Belgian context in agreement with stakeholders. The first main objective of this pilot study was to evaluate the implementation process of SPIRIT in the Belgian context, thereby gaining a deeper understanding of the barriers and facilitators to the implementation, as recommended by Diestelkamp et al., who conducted a systematic review on interventions for adolescents and young adults following alcohol-related events in emergency care in 2016 [27]. Diestelkamp and his colleagues concluded that process evaluation analyses were rarely performed, and when performed, in a heterogeneous way, making it difficult to generalise findings. Also, the investigation of moderators and mediators of effectiveness was mostly missing. Therefore, they recommend including a more standardised and comprehensive approach to implementation evaluation when analysing these types of interventions. In this study, a standardised implementation evaluation was therefore conducted, guided by the Consolidated Framework for Implementation Research (CFIR) [28, 29]. The second main objective was to conduct a preliminary effectiveness analysis on decreasing problematic alcohol use, preventing recurrent AAIs, strengthening parent-child interaction and parenting skills, and detecting and treating potential underlying psychological disorders among SPIRIT participants. Finally, as this study aimed to create a change by implementing a newly adapted program that could generate health and behavioural changes for participants, it was found essential to guide the implementation process by participatory action research (PAR). Including stakeholders, as is recommended by PAR, can increase the success of the implementation [30]. Moreover, using PAR allowed us to get a thorough insight into moderators and mediators in the process of change [30]. In this article, we first describe the adaptation process of SPIRIT and the evaluation of the SPIRIT implementation process (guided by CFIR and PAR). Subsequently, the preliminary analysis of the program’s effectiveness are presented.

## Methods

### Study context

The SPIRIT is a multidisciplinary follow-up treatment program for adolescents with AAI, which was adapted, pilot-tested and evaluated in this SPIRIT pilot study over the period 2021–2025. The study took place in the city of Antwerp, located in the Flemish region of Belgium, Europe. Antwerp is the second largest city in Belgium, with 565,700 inhabitants as of 2025 [31]. The University of Antwerp developed the SPIRIT in collaboration with the Reinier de Graaf Hospital in Delft (the Netherlands), the Antwerp-based hospital ZAS (‘Ziekenhuis aan de stroom’) Augustinus and the Antwerp University Hospital (UZA). The foundation of the program was based on the already existing and similar program ‘Jeugd en Alcohol’ in the Netherlands [22, 26], but adjusted to the Belgian context in agreement with stakeholders, including the initiator of the Dutch outpatient clinic and author of this article Prof. Nico van der Lely.

### Study design

This study used a mixed-method approach. The adaptation of the Dutch program ‘Jeugd and Alcohol’ to SPIRIT and the implementation process of SPIRIT followed the stepped approach of PAR. PAR was considered a suitable framework, as it focuses on creating change and gaining practical knowledge through stakeholder involvement [30]. The involvement of stakeholders was considered essential, to be able to adapt the program to the needs of the stakeholders and therefore, enhancing the chance of success. PAR encompasses the cyclical process of mapping stakeholders’ needs, analysing the context, and developing and implementing the intervention according to the needs and context. This was then followed by an evaluation with stakeholders and adaptation of the intervention based on this evaluation [32]. Additionally, the CFIR was utilised for the evaluation of the adaptation and implementation process. CFIR is one of the most frequently used determinant frameworks to assess contextual factors influencing implementation [28]. Based on multiple published implementation theories, the framework comprises various constructs organised around five major domains: innovation, outer setting, inner setting, individuals, and the implementation process [28, 29]. Next to these domains, the use of an outcome addendum has been proposed recently [33]. We applied the CFIR framework to gain a comprehensive understanding of the contextual factors influencing the implementation of the SPIRIT in the local context of Antwerp [34]. This was done by, amongst others, interviews and brainstorming meetings with stakeholders. Finally, the preliminary effects of the SPIRIT were investigated using a quasi-experimental design [35], with pre- and post-measurements among patients aged 10–17 participating in the intervention, without including a control group or randomisation. Therefore, the participants served as their own control. In this methodology section, the process of developing and implementing the SPIRIT is outlined first. Second, the implementation process methodology is explained, followed by the methods used for the preliminary effect analysis.

### Implementation process

#### Preparatory phase

First, we analysed the context in which the SPIRIT would be implemented. In this regard, it was necessary to gain knowledge of the current standard care for adolescents with AAI in Antwerp/Belgium. To answer the research question: ‘How is the care for adolescents below 18 years old with acute alcohol intoxication organised in Belgium at the starting point of the study?’, both an online survey with paediatricians in Flanders and semi-structured interviews with paediatricians in Antwerp were conducted. The survey was conducted among Flemish paediatricians in September 2021 and disseminated through the Flemish Network of Paediatricians (*n* = 23). The semi-structured interviews were conducted with the heads of paediatric departments of the hospitals in the city of Antwerp by the main researcher of the team (HvR) in 2021 (*n* = 4). The interviews were held in Dutch, lasted around 30 minutes on average and were recorded via MS Teams or telephone (see supplementary file 1 for the semi-structured interview scripts).

Additionally, we identified stakeholders (*n* = 57) and mapped their views on the intervention and their willingness to engage. Table 1 describes the number of included stakeholders, their profession and their level of experience.

**Table 1:** Overview of included stakeholders, their profession, organisation, number and level of experience in the spirit pilot study

Stakeholder’s profession (organisation)	Number of stakeholders	Level of experience
Researchers (University of Antwerp, background in medicine, paediatrics, psychology and sociology)	1	Junior
	5	Senior
Paediatricians (UZA and ZAS)	3	Senior
Head of Paediatrics department (UZA, ZAS and AZ Monica)	6	Senior
Psychologists (ZAS, CGG Vagga, RdGG)	3	Senior
Emergency physicians (ZAS)	1	Junior
	12	Senior
Head of Emergency Department (ZAS)	2	Senior
Senior function at Department of Mental Health Care for Children and Adolescents (ZAS)	2	Senior
Registrars Paediatrics (UZA)	20	Junior
IT employees (University of Antwerp, ZAS)	2	Senior
Director (VAD)	1	Senior

ZAS, ‘Ziekenhuis aan de Stroom’, Antwerp-based hospitals

CGG Vagga, Centre for Mental Healthcare Vagga, Antwerp

RdGG, Reinier de Graaf Hospital, Delft, the Netherlands

VAD, Flemish centre of expertise on Alcohol and other Drugs

UZA, Antwerp University Hospital

Brainstorming sessions were than organised with these stakeholders, for instance the researchers of the team (authors HvR, IG, SV, JDD, NvdL and GVH), the multidisciplinary academic advisory committee of the study (consisting of the researchers of the team and experts from the field) and field partners (including the authors LV, BDT, AVA, EV, FDM, MM), respectively. Additionally, observations of the outpatient clinic at Reinier de Graaf Hospital in Delft (the Netherlands), where the Dutch program ‘Jeugd en Alcohol’ has been initiated, were organised. Notes were taken during each meeting, including the field observations, which were subsequently analysed qualitatively for the implementation process evaluation, of which the details are explained in further detail under ‘implementation process evaluation’ in this method section. During these meetings the design, context and implementation process of SPIRIT were discussed, so that potential barriers to implementing the SPIRIT intervention could be identified, and strategies to overcome these barriers were developed through consultation with the stakeholders.

#### SPIRIT intervention development

The SPIRIT was based on the existing and effective outpatient clinics for adolescents with AAI in the Netherlands (‘Jeugd en Alcohol’) [22, 36]. However, this foundation was adapted to the visions of important stakeholders, to the specific Belgian context and the identified barriers, which were all assessed in the preparatory phase of the SPIRIT pilot study. This resulted in the SPIRIT program.

**Fig. 1:**
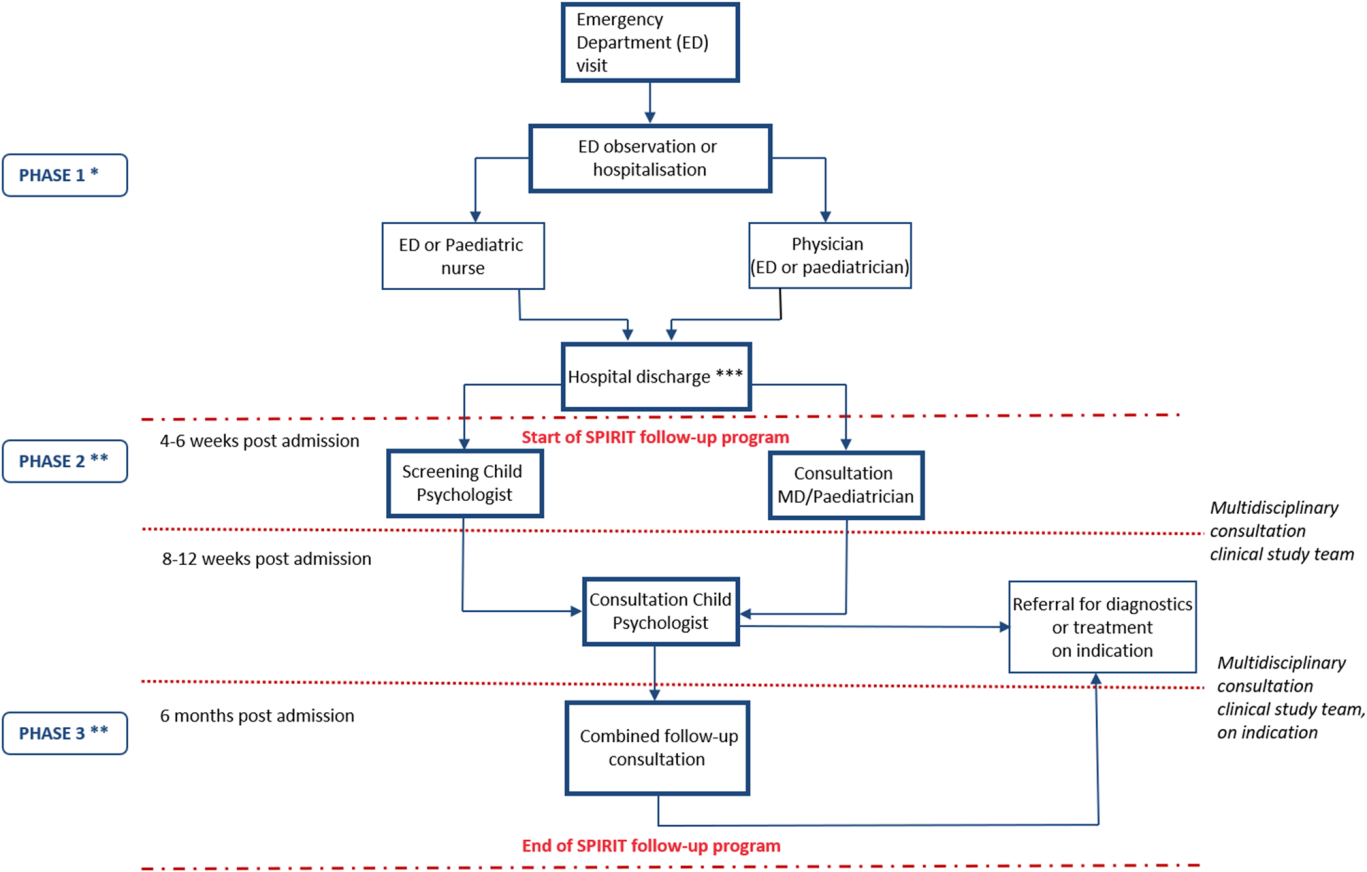
Flowchart of adolescents with acute alcohol intoxication from arrival at the emergency department to completion of the SPIRIT intervention. *Phase 1 was performed in the hospital where the adolescent with AAI was admitted. ** Phases 2 and 3 were performed in the SPIRIT follow-up program at ZAS Augustinus in Antwerp. *** Moment of inclusion in the SPIRIT study

The schematic flow chart of SPIRIT is shown in Fig. 1. SPIRIT was designed as a 6-month outpatient follow-up program for adolescents aged 10–17 years who presented with AAI at the emergency, paediatric, or intensive care departments of the hospitals ZAS Augustinus, ZAS Vincentius, or UZA. The inclusion criteria included a clinically diagnosed or laboratory informed (increased blood alcohol concentration, BAC) alcohol intoxication. Additional criteria included having decreased consciousness (Glasgow Coma Scale, GCS, ≤14) and/or an indication for an overnight admission to hospitalisation. In the case of direct referral for addiction treatment, patients were not included in the study. Eligible participants and their parents and/or guardians provided written informed consent before enrolment. At discharge (T0), participants and their parents received a QR code linking to a study-specific website [37]. This website guided participants through several steps. First, participants completed a self-test on alcohol use based on the Alcohol Use Disorders Identification Test (AUDIT) questionnaire [38], with personalised feedback based on their responses, developed by the Flemish Expertise Centre for Alcohol and other Drugs (VAD). Second, they filled in a baseline survey (T0 survey) developed by the research team, which included questions on socio-demographics, medical background, alcohol use, drug use, circumstances of alcohol intoxication, psychological symptoms (assessed via the Child Behaviour Checklist (CBCL) for parents and Youth Self Report (YSR) for adolescents [39]), parent-child interaction (Conflict Behaviour Questionnaire (CBQ) [40]), parenting style (for parents only, assessed using the Parenting Scale (PS) [41]), and how they experienced the hospitalisation. This hospitalisation experience was assessed by asking how the participants and their parents rated the hospitalisation on a scale from 0 to 10, by asking open questions regarding suggested improvements for hospitalisations and aspects they appreciated about the hospitalisation, and by presenting various statements about the hospitalisation, for instance ‘I felt save to share my story with the emergency physician’, which could be answered using a 5-point Likert Scale, ranging from ‘totally disagree’ to ‘totally agree’. Finally, participants and their parents received educational materials on alcohol misuse from resources of the VAD and Trimbos Institute (The Netherlands).

At T0, the treating physician also completed a survey regarding the medical characteristics of the participants’ admission in the Electronic Health Record (EHR). Next, the physician organised the referral to the SPIRIT, either via the EHR or via a pseudonymised email to the clinical team (HvR and LV).

Four to six weeks post-discharge, participants and their parents were invited for screening consultations at the Department of Paediatrics at ZAS Augustinus (T1). These consultations included sessions with a psychologist for the participant and parents separately (LV) and joint sessions with a medical doctor (HvR, AVA or EV). The psychologist conducted psychological screening, psycho-education and motivational interviewing. The medical doctor focused on medical screening, psycho-education and motivational interviewing. Four to six weeks after the T1 consultations, adolescents and their parents attended a second consultation with the psychologist, where advice based on the initial screening was provided. Motivational interviewing and brief cognitive behavioural therapy, when indicated, were used.

At six months post-admission (T2), a final follow-up consultation with both the psychologist and the medical doctor was conducted, to assess progress and, if necessary, provide a referral. Participants and their parents completed a follow-up survey (T2 survey) via the study website [37]. This survey repeated the baseline questions and included additional items about recurrent AAI admissions and participant experiences with the follow-up program.

#### Intervention implementation

In May 2023 the intervention was implemented, starting with eligible patients from ZAS Augustinus and ZAS Vincentius. From December 2023, patients from UZA could also be included in the SPIRIT. In October 2024, the inclusion of participants stopped, and the final T2 measurement was finished in March 2025. In total, 14 patients were included in the SPIRIT pilot study.

During the implementation phase of the study, qualitative data was obtained from various meetings with stakeholders, namely monthly research meetings (with authors HvR, IG, SV, JDD, NvdL, GV), monthly clinical meetings (including authors HvR, IG, LV, AVA, EV, NvdL) and brainstorming sessions with other stakeholders (including authors BDT, MM). In these meetings, the research team reflected on the progress and jointly tackled emerging problems in the intervention implementation. The intervention protocol was then adjusted accordingly, in consultation with important stakeholders. For example, we allowed for follow-up consultations to be performed by telephone instead of in person when requested by participants and their parents. The other adjustments will be elaborated on in the results section of this article.

### Implementation process evaluation

The CFIR framework was used to evaluate the implementation process and report the implementation outcomes of the implemented intervention [28, 33]. The primary outcome was the identification of barriers and facilitators to implementing the SPIRIT.

#### Data collection

Table 2 provides an overview of all data collection methods employed in this study, both for the implementation process evaluation and the preliminary effect analysis (see ‘Preliminary effect analysis, data collection’ for more details on the latter).

**Table 2:** Overview of data collection methods (including the qualitative or quantitative nature of data) connected to the research objectives of the SPIRIT pilot study

Research outcome	Data collection methods
Assessing barriers and facilitators for implementation (qualitative data)	Survey paediatricians (quantitative data)
	Interviews heads of paediatric departments
	Brainstorming sessions with the researchers of the team
	Brainstorming session with the academic advisory committee of the study
	Brainstorming sessions with stakeholders
	Observations outpatient clinic Delft
	Monthly clinical meetings
	Monthly research meetings
	T0 survey adolescents (partly)
	T0 survey parent(s) (partly)
	T2 survey adolescents (partly)
	T2 survey parent(s) (partly)
Preliminary effect analysis (quantitative data)	T0 survey physician
	T0 survey adolescents (partly)
	T0 survey parent(s) (partly)
	T2 survey adolescents (partly)
	T2 survey parent(s) (partly)
	Number of eligible patients during the study period

As mentioned above, multiple data collection methods were used to analyse the context in which the intervention would be implemented. In addition, the following data sources were used for assessing barriers and facilitators to implementation: 1) notes from the (brainstorming) sessions with the research team, 2) notes from brainstorming session with the academic advisory committee of the study, 3) notes from brainstorming sessions with stakeholders, 4) notes from clinical meetings, and 5) questions from T0 and T2 surveys of participants and their parents regarding their experiences of and opinions about the intervention. To be able to perform qualitative document analyses, notes were taken during these meetings, including the monthly research meetings, which were subsequently qualitatively analysed, as explained further in the next section ‘data analysis’.

#### Data analysis

Quantitative data deriving from the paediatric survey were analysed using descriptive statistics. These were performed in IBM SPSS Statistics, version 29.0 (Armonk, NY, USA: IBM Corp.). The semi-structured interviews with paediatricians were transcribed using Whisper-Zero (Gladia), and errors were subsequently checked (HvR). Of all the other qualitative data collection methods described in Table 2, the notes of these meetings and/or observations were exported into NVivo software version 14.23.4 for analysis. The same accounts for the qualitative data deriving from the participant surveys. Triangulation of data from these different study phases was then conducted. Subsequently, the qualitative data documents were read several times to familiarise with the data (HvR). The data was then coded systematically following the Framework Method [42] (HvR). To achieve this, a thematic framework was developed by the research team based on the existing codebook of CFIR for NVivo (shared by the CFIR team with the SPIRIT researchers) and complemented with themes emerging from the transcripts (HvR, IG, GVH). The codes were translated to English and then charted and rearranged in Microsoft Excel (for Microsoft 365 MSO version 2402), so the thematic content would match CFIR domains and constructs. To report the findings, the included CFIR constructs were assessed as factors influencing implementation, relating to (1) barriers to implementation and (2) facilitators to the implementation of the intervention. As no guidelines for assessing barriers and facilitators exist within the CFIR framework, this was carried out in a neutral way (counting and weighing the amount of negative, neutral and positive influencing factors within every construct) and in mutual agreement between authors HvR, IG and GVH, who have a multidisciplinary background in medicine, psychology and sociology.

### Preliminary effect analysis

#### Outcome variables

The primary outcome of the effect analysis was problematic alcohol use over the past 12 months (baseline) or past 6 months (follow-up), as measured by the AUDIT-C score. The AUDIT-C is a shortened version of the validated AUDIT questionnaire developed by the World Health Organization [38]. It is also validated for screening problematic alcohol use in adolescents [43]. The score ranges from 0 to 12, where zero indicates non-drinking and 12 indicates unhealthy alcohol consumption behaviour. A cutoff score of ≥ 3 has been recommended for the detection of problematic alcohol use among adolescents [44]. Additionally, various secondary outcomes were assessed. Secondary outcomes included recurrent AAI since study involvement (yes/no), and the frequency of binge drinking at baseline (in the last 12 months) and follow-up (in the last 6 months). The definition of binge drinking used was the consumption of 4 standard alcoholic glasses in 2 hours for females and 6 standard glasses for males [3]. Furthermore, internalising and externalising problems were assessed based on a (sub)clinical score on the internalising, externalising and/or total problem scale of the CBCL and/or YSR [45]. Parental rules regarding the alcohol use of their child were assessed among parents using the question, ‘Do you permit your child to drink alcohol below 18 years old’? (always/only above 16 years old/on special occasions/never). The parent-child interaction was measured with the CBQ, which questions interactions and problems in the relationship between adolescents and parents, with a score ranging from 0 to 20, where zero represents a low degree of conflict behaviour, and 20 represents a high degree of conflict behaviour [40]. Parenting style was measured by using the parents’ score on the PS, which ranged from 0 to 7, where 0 indicates functional parenting and 7 represents dysfunctional parenting [41]). Moreover, the perceived effect on parent-child interaction and alcohol use was measured with the 5-point Likert scale score for the statements ‘The follow-up trajectory of the outpatient alcohol clinic improved the relationship with my child/parents’ and ‘The follow-up trajectory of the outpatient alcohol clinic made me drink less alcohol’ (totally disagree/disagree/agree/totally agree/no opinion). Although the SPIRIT did not target smoking and drug use directly, these variables were questioned to measure a potential additional effect. Therefore, smoking (no/yes, but I stopped/yes, currently), cannabis use (no/yes, but I stopped/yes, currently) and other illegal drug use (no/yes) were measured at both baseline (over the last 12 months) and follow-up (over the last 6 months) among the adolescents. Finally, the reach of patients was assessed by comparing the number of participants with the total amount of admitted eligible patients during the study period based on laboratory data (patients with a positive BAC).

#### Patient characteristics

Sociodemographic variables, namely age, sex, type of education, living situation, and family position, were measured at baseline and follow-up. Clinical characteristics included the BAC in g/L, the performance and results of urine drug screenings, GCS scores (0–15), type of transport to the ED and medical history. Additionally, previous alcohol incidents were measured at both baseline and follow-up. Also, risky alcohol behaviour and negative alcohol experiences at baseline were measured with an adapted version of the CRAFFT 2.0 (Car, Relax, Alone, Forget, Friends, Trouble) [43], in which only questions regarding alcohol use were included and not regarding other drug use, with the editor’s consent. Aspects that are asked within this questionnaire are, for instance, if participants ever drank alcohol when they were alone, or with the goal to feel more relaxed.

#### Data collection

Participant demographics were assessed during or shortly after admission through surveys administered to participants and parent(s) (T0). At the same time point, clinical characteristics of the AAI admission were obtained from the treating physician via the hospital charts (T0). Primary and secondary outcomes for both participants and parents were assessed using surveys at T0 and again 6 months post-admission (T2). These T0 and T2 surveys were developed by the research team. Finally, the total number of admitted 10–17-year-old patients with AAI during the study period was assessed by the laboratories of the included hospitals. They screened patients who met our inclusion criteria and had a positive BAC ( > 0.1 g/L at the ZAS hospitals and > 0.03 g/L at UZA) during our study period. See also Table 2 for an overview of the data collection methods.

#### Statistical analyses

Demographics and clinical characteristics were analysed using descriptive statistics to identify baseline characteristics of the study population. Categorical variables were expressed as proportions, and numerical data were presented as mean (Standard Deviation, SD) or median [interquartile range, IQR], depending on the distribution of the variables (based on histogram assessment and Kolmogorov–Smirnov tests). Intervention effects on primary and secondary outcomes were assessed using the Wilcoxon Signed Ranks test for paired ordinal data and the McNemar test for paired nominal data. The significance level for all statistical tests was set at 0.05. Statistical analyses were performed in IBM SPSS Statistics, version 29.0 (Armonk, NY, USA: IBM Corp.).

## Results

### Implementation process evaluation

**Fig. 2:**
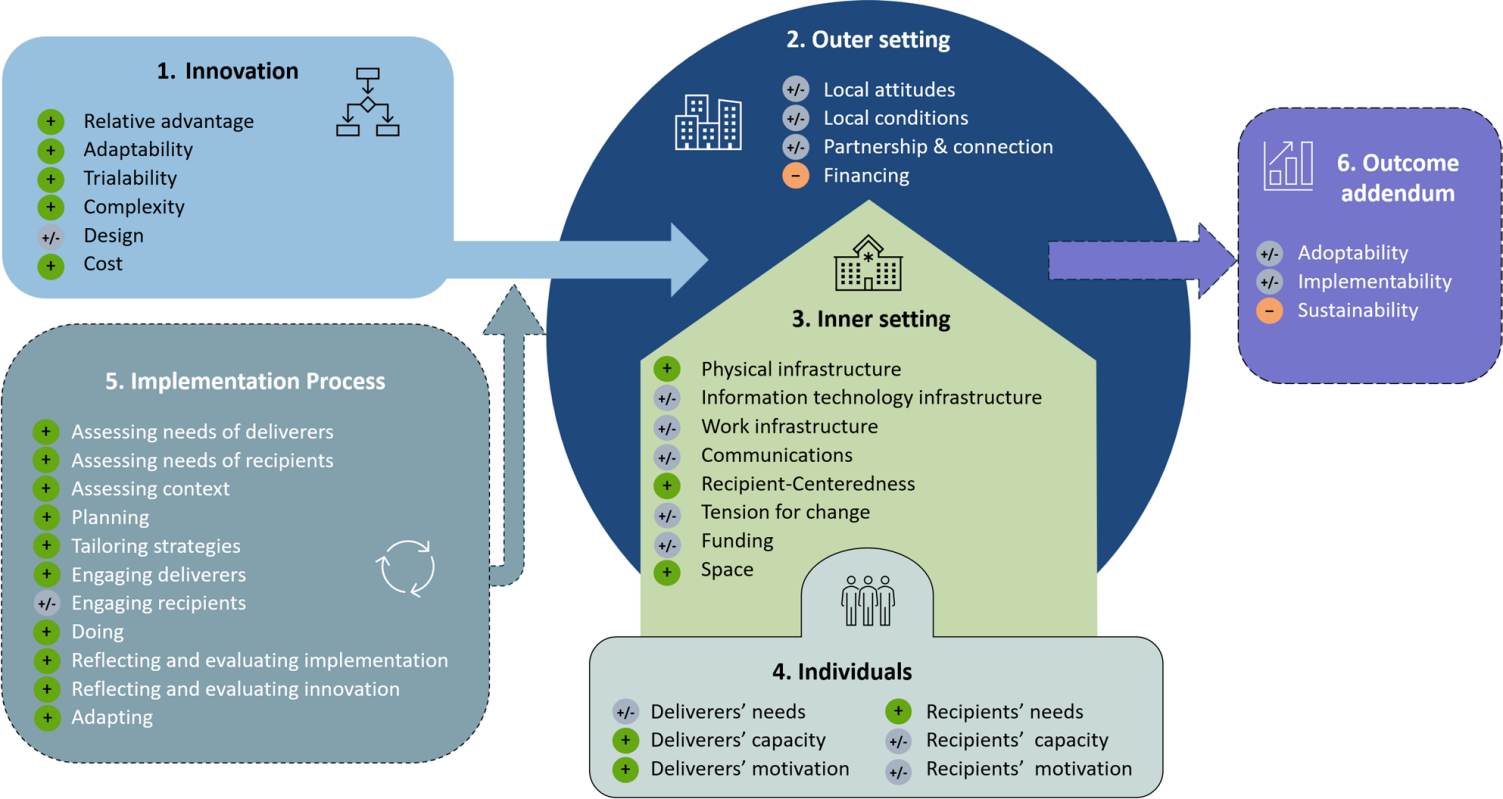
The interpretations of the influence of the 38 relevant constructs from CFIR domains on the implementation of the SPIRIT. Figure based on Damschroder et al. [28, 33] and the Center for Implementation [46]

The implementation process was described by assessing barriers and facilitators to implementing the SPIRIT across 38 relevant constructs of the 5 CFIR domains and the outcome addendum. Figure 2 provides an overview of the included constructs and indicates whether these constructs were considered barriers or facilitators to the implementation of the intervention. Of the in total 38 included constructs, 2 were considered as barriers (-), 21 as facilitators (+) and 15 as both barriers and facilitators (±). The themes and supporting quotations from the qualitative analyses, connected to the corresponding CFIR constructs and domains, are presented in detail below.

#### Innovation

##### Relative advantage (+)

Before the start of the SPIRIT, follow-up treatment for adolescents with alcohol intoxication was not embedded in the Belgian healthcare system. A treatment protocol for these patients was absent; paediatricians and child psychologists were rarely involved, and often, patients were not referred to a paediatrician or other medical specialists. As stated by one of the paediatricians: *‘The SPIRIT intervention could be very relevant for patients with a risk of addiction, who do not get a standardised follow-up treatment at the moment’ [Paediatrician 3].* Furthermore, it was believed by the psychologists and research team that the SPIRIT could assist in engaging parents, which was not yet done in a standard manner.

##### Adaptability (+)

The research team decided to implement SPIRIT in various stages, with PAR guiding the evaluation and adaptation throughout the process, in close collaboration with stakeholders.

##### Trialability (+)

In collaboration with stakeholders, the already existing program ‘Jeugd en Alcohol’ in the Netherlands was adjusted to the Belgian context, resulting in SPIRIT. Together with stakeholders, the research team decided to conduct a pilot study to validate the implementation of the intervention in the Belgian context. Nevertheless, when serious (implementation) problems would have occurred during implementation, it would have been possible to stop or pause the pilot study in consultation with the stakeholders.

##### Complexity (+)

The intervention comprised multiple components, and therefore, it was considered a complex intervention by the research team. Consequently, the researchers chose to conduct a pilot study instead of a randomised controlled trial.

##### Design (±)

Patient inclusion materials, such as informed consent forms and an information leaflet, were stocked at the EDs. These included a QR code, which led participants and their parents to a website with alcohol education materials. Almost all parents (*n* = 13) found the information leaflet about the SPIRIT useful. However, most participants did not find the educational video on youth and alcohol (developed by Jellinek in the Netherlands) appealing. On the other hand, most participants stated they had learned something from the ‘Drink, Drank, Drunk’ leaflet developed by the VAD. Furthermore, participants rated the overall innovation a 7.1/10 and parents an 8.4/10. The majority found the consultations with the psychologist and medical doctor to be useful (*n* = 9) and felt listened to during these sessions (*n* = 11). One parent stated: *‘The consultations were clarifying.’[Parent 8]* Other parents said: ‘*The staff was very empathetic and professional’ [Parent 18]* and *‘[I appreciated] the neutral attitude’ [Parent 19].* Some parents advised setting up a more in-depth conversation about alcohol at the time of discharge: *‘It would be good if the emergency doctor would give more education about the severity of the situation’ [Parent 8].* And: *‘[I would recommend to] already set up a consultation with an expert at the time of discharge at the emergency room’ [Parent 6].*

##### Cost (+)

The costs related to the innovation could be divided into direct implementation and service costs (psychologist working costs, medical doctor and/or paediatrician working costs, and the participants’ co-payments for psychological consultations (€180 per patient) that were covered) and indirect implementation costs (time dedicated by researchers and stakeholders, time dedicated by IT workers, and staff training). The direct costs were partly covered by ZAS Augustinus, and partly by the Research Chair ‘Reinier de Graaf Youth and Alcohol’ at the University of Antwerp. As a result, participants were not charged for the follow-up intervention. However, costs were associated with the initial ED admission, which was not part of the intervention. Therefore, although the researchers advised performing a urine drug screening for all patients to avoid missing cases of comorbid drug use, this was not always carried out by emergency physicians, as patients were required to pay for this screening themselves. In addition, the indirect costs of the implementation were covered by the particular organisations the researchers and/or stakeholders were affiliated with.

#### Outer setting

##### Local attitudes (±)

On the one hand, paediatricians believed the SPIRIT to be important and beneficial for specific patients because no standardised and child-friendly protocol had been in place before. On the other hand, one paediatrician also mentioned the risk of stigmatising: *‘I think a big part of this problem is experimental behaviour, which is normal behaviour in puberty. I wonder if you will not stigmatise these patients’ [Paediatrician 3].* Additionally, another paediatrician stated that *‘Most paediatricians are not engaged in the topic of alcohol intoxications among adolescents because they do not often come across this diagnosis in the hospital at the moment’ [Paediatrician 4].*

##### Local conditions (±)

Two different hospital groups exist in the city of Antwerp, and referring patients from one hospital group to the other is not common. However, according to the heads of the various paediatric departments, this would not have been a problem among paediatric patients due to the small number of patients in this pilot study. The paediatricians (*n* = 4) also mentioned that alcohol use among adolescents is considered normal in society, and a shift in political and societal mindset is therefore needed. This is also demonstrated by the legal age limit for purchasing alcoholic beverages (specifically beer and wine) in Belgium, which is 16 years. Furthermore, an alcohol screening and follow-up program for adults specifically was recently set up in ZAS Augustinus with the help of a federal subsidy. In this way, a preventive approach to alcohol-related issues already existed in this hospital.

##### Partnership & connections (±)

The overarching organisation of paediatricians in Belgium decided recently to make the prevention of paediatric diseases more of a priority than before; therefore, the SPIRIT aligned with their vision and was considered relevant. Additionally, according to the multidisciplinary academic advisory committee of the study, close collaboration with all stakeholders, including paediatricians, emergency doctors, psychologists, and politicians, was deemed important. However, psychologists were concerned about the long waiting lists in child psychology centres, which could pose a problem for participants in need of a referral for additional care. To tackle this problem, a fixed contact person was made available at CGG Vagga, a specialised centre for mental healthcare in Antwerp, for consultation and referral arising from the SPIRIT, and a short waiting time was guaranteed for participants in need of (pre-)addiction treatment.

##### Financing (-)

No external funding was available for this project, as medical psychologists do not receive funding for diseases without a medical convention, which does not currently exist for alcohol intoxication in Belgium. Moreover, despite multiple attempts to apply for a federal grant, no federal grant was awarded to this project.

#### Inner setting

##### Physical infrastructure (+)

A decision was made to locate the SPIRIT follow-up program at ZAS Augustinus due to its geographical location in Antwerp. Combined consultations were also more feasible for participants and their parents in this hospital, given that the Department of Mental Health for Children and Adolescents and the Department of Paediatrics are located next to each other.

##### Information technology infrastructure (±)

Emergency physicians at ZAS Augustinus and ZAS Vincentius could refer a patient to the SPIRIT by a build-in ‘order’ in the EHR. This order was received by the child psychologist (LV), who would then phone the participant’s parents to plan the consultations. During the implementation of SPIRIT, the psychologist and medical doctors reported that participants’ contact details were sometimes missing in the EHR, making it difficult for participants to be easily reached. For this reason, emergency physicians were asked by the research team to always check the contact details of the participants in the EHR from then on. Additionally, emergency physicians at all hospitals had to complete a brief questionnaire with an ‘auto text’ shortcut in the EHR. Moreover, to overcome the potential barrier of missing participants, emergency physicians advised the researchers to build a tracker for eligible patients in the EHR so that potentially missed patients could still be included. However, the ethics committee did not provide approval to contact patients who had already been discharged and this change could not be implemented. For this reason, missed cases were tracked retrospectively for data collection purposes only (e.g., assessing the reach of the SPIRIT). Finally, emergency physicians and parents both reported that the QR code used for referral to SPIRIT worked easily.

##### Work infrastructure (±)

Because time is often scarce for emergency physicians due to their demanding clinical tasks, the additional tasks involved with participation in the SPIRIT needed to be limited. That is why we aimed to achieve a user-friendly and efficient work infrastructure. The physicians were satisfied with the referral order in the EHR. However, the ‘auto-text’ questionnaire was often overlooked. In those cases, the clinical information needed to be extracted from the EHR notes. Because UZA had a different EHR than the ZAS Hospitals, they could not work with the build-in order for referral and had to email the medical doctor instead, which was more time-consuming.

##### Communications (±)

A ‘team page’ was created in Microsoft Teams for communication between the research team and the emergency physicians of the ZAS hospitals, as well as to discuss issues related to participant eligibility. Both parties were satisfied with this process. However, such a communication tool did not exist between the emergency physicians of UZA and the research team. There, regular communication was undertaken directly between the clinical leader (emergency paediatrician, MM) and the researchers.

##### Recipient-centeredness (+)

The decision was made to organise the consultations in the outpatient Department of Paediatrics and the Department of Mental Health for Children and Adolescents of the ZAS Augustinus instead of in a psychology or addiction clinic. In this way, the focus was more on the somatic component of alcohol intoxication, which would potentially lower the chance of stigmatisation and because of that, the researchers expected a lower threshold for patients to participate. Additionally, in this way, we could prevent additional costs for participants.

##### Tension for change (±)

Most paediatricians cited personal reasons when describing the need or an intervention like SPIRIT. One paediatrician said:*‘Alcohol intoxications among adolescents are [an] important [topic]’ [Paediatrician 1]. A*nother paediatrician said: *‘I hear stories via my children about their friends who get into accidents or need hospital monitoring due to alcohol intoxications. I have also experienced social pressure among my children to drink [alcohol] together with friends before going out. One of their arguments is the high price setting of alcoholic drinks in social venues. I have also experienced more alcohol use in youth movements, I wonder if social pressure plays a role here. I think it is important to screen for psychological diagnoses in these types of patients. That is why I am interested in this research’ [Paediatrician 3].* Another common theme guiding emergency physician’s belief in the need for an intervention like SPIRIT was that they had seen many admissions due to intoxication among adolescents and young adults. Additionally, one physician suggested that: *‘We do not have a standardised protocol or working agreements for this group of patients at the moment, and we would like that’ [Stakeholder 4].* On the other hand, some emergency physicians had doubts about the expertise of paediatricians and psychologists in the follow-up treatment of patients with alcohol intoxication. Some physicians also communicated some hesitation about the demanding workload that emergency physicians already have to manage. This was one of the reasons why the inclusions in UZA were done by emergency paediatricians or paediatric residents instead of emergency physicians. Some parents also mentioned the need for change. For instance, one parent said: *‘I had the feeling that the health care professionals at the emergency room developed an increased tolerance [for alcohol intoxications among adolescents]. It happens too often; therefore, it is considered normal [by the health care professionals]’ [Parent 23].*

##### Funding (±)

No long-term funding was available for the project. However, the pilot study was covered by two short-term funding sources: the co-payment of participants was covered by the University of Antwerp research chair ‘Reinier the Graaf Youth and Alcohol’. ZAS Augustinus paid the psychologist’s working costs within the SPIRIT. Funding for additional psychological examinations was not available. For the program to be implemented in a sustainable way, long term funding to cover the direct implementation and service costs (e.g. psychologist and medical doctor/paediatrician working costs, co-payment of participants) would be needed.

##### Space (+)

In all SPIRIT contexts where patient contact was needed, the appropriate consultation rooms were available (both at the emergency room and the outpatient clinic). Moreover, as the participant surveys were available on a mobile phone, participants and their parents were able to finish these surveys at a time and space that suited them: in the emergency room, at home or in the waiting room of the outpatient clinic.

#### Individuals

##### Deliverers’ needs (±)

At the start of the implementation, resistance among some emergency physicians was noted due to concerns about the additional and potentially time-consuming work. Therefore, the time commitment required to achieve SPIRIT tasks was minimised for emergency physicians.

##### Deliverers’ capacity (+)

It was decided that only one or two medical doctors and/or paediatricians would be involved in the SPIRIT, allowing them to become experts on the topic of AAI among adolescents in Antwerp. The involved medical doctors and paediatricians reported back that this capacity was indeed achieved. The psychologist and medical doctors who were involved in the consultations were trained in motivational interviewing, either via their psychological background training or by following the recent course ‘motivational interviewing’ by the VAD. Additionally, clinical meetings with the involved medical doctors and psychologists were organised monthly to discuss all patients anonymously. Specific directions for motivational interviewing, diagnostics and/or treatment were discussed here, which was found helpful by the deliverers.

##### Deliverers’ motivation (+)

Based on various brainstorming sessions with stakeholders, the emergency doctors, psychologists, and paediatricians from the ZAS hospitals, as well as the emergency paediatricians and paediatric residents from UZA, were enthusiastic and motivated about the innovation.

##### Recipient’s needs (+)

From the field observations of the follow-up program in Delft (the Netherlands), conducted by researcher HvR, it appeared that some adolescents who experience acute alcohol intoxication consume alcohol regularly due to coping reasons or are at risk of alcohol addiction (based on assessments from stakeholders from the follow-up program in Delft). Therefore, they are in need of additional help, which could be organised via the SPIRIT. The stakeholders from the follow-up program in Delft had the experience that there are also patients who belonged to a community in which frequent drinking is normalised. As SPIRIT was designed to address these types of needs, the researchers believed that patients and their parents would benefit from the education and motivational interviewing offered by the SPIRIT. Also, adolescents with AD(H)D or other psychological disorders are prone to drinking alcohol more frequently, as was experienced by the psychologist and paediatricians at the follow-up program in Delft. SPIRIT allows for screening of these types of comorbidities. Furthermore, some participants also indicated that they did not learn anything from the ED admission itself (1 participant) or that nothing changed at home after the admission only (8 participants). Also, parents indicated the need for the intervention: *‘[There is a need for] better follow-up after emergency admission’ [Parent 20],* and *‘I agree with the (scientific) attention for this important topic’ [Parent 24].* Another parent stated after following the intervention: ‘*I was surprised by how little my son knew about the effects of alcohol’ [Parent 19].*

##### Recipient’s capacity (±)

When participants or parents were unable to complete the online questionnaires at home, they were asked to do so in the outpatient department waiting room on one of the available tablets. The education materials and questionnaires were in Dutch, which could have been a problem for non-Dutch-speaking participants and/or parents, as was raised as an issue by one parent. For some participants, difficulties arose due to divorced parents, as stated by the following parent: *‘Answering some of the questions [of the survey] is difficult for me because my child is living with her mother’ [Parent 22].*

##### Recipient’s motivation (±)

As suggested by the emergency physicians and psychologists, there might be shame among recipients, leading to a barrier to participation. These stakeholders also raised their concern regarding the group of 16–17-year-olds, who potentially have more resistance to a consultation with a paediatrician/child psychologist due to their older age. Signing the informed consent forms may have been a barrier to participation for some patients, as suggested by the psychologists and research team. Among the participants’ parents, approximately 2/3 were already motivated to participate in the follow-up program, whereas the other 1/3 needed to be motivated by the psychologist when she telephoned them to plan the consultations.

#### Implementation process

##### Assessing needs of deliverers (+)

Interviews with paediatricians were conducted, and brainstorming sessions with researchers and stakeholders in the field were organised.

##### Assessing needs of recipients (+)

Lessons learned from similar follow-up programs in the Netherlands, with 18 years of experience, were considered. Also, the surveys were pilot-tested by the researchers and adolescents (children of the researchers from the targeted age group). Furthermore, the initial ED admission and follow-up program were evaluated through surveys conducted with participants and their parents. Finally, the research team had sufficient experience in working with adolescents with alcohol-drinking problems and/or other risk-seeking behaviour, both clinically and in research.

##### Assessing context (+)

The context was assessed through interviews with the heads of paediatric departments in Antwerp and a survey of Flemish paediatricians. Observations of a similar follow-up program in Delft, The Netherlands, were also organised.

##### Planning (+)

The researchers planned to initiate the innovation using existing information leaflets and materials, with the goal of evaluating and adapting these materials as needed. Additionally, it was decided to conduct a pilot study of the SPIRIT within a limited timeframe. Finally, the enrolment of patients first started in ZAS Vincentius and ZAS Augustinus, followed by UZA seven months later.

##### Tailoring strategies (+)

By using PAR, obstacles that arose along the way were addressed. For instance, 16- and 17-year-olds are normally not seen by paediatricians in Belgium for follow-up consultations, but an exception was made for this pilot study. Moreover, the initial idea was to develop a phone application for participants and their parents to share information materials and the surveys. However, a website was developed instead due to insufficient budget.

##### Engaging deliverers (+)

The research team had decided to involve local paediatricians and emergency physicians in the implementation process. When issues regarding inclusions arose, emergency physicians were able to communicate them to the research team via the Microsoft Teams channel, as proposed by the head of the emergency department. Additionally, in consultation with the involved head of the emergency department and paediatricians, it was decided to let the researchers present the project to emergency physicians and paediatric residents at the start of enrolment in an interactive manner.

##### Engaging recipients (±)

We did not organise focus group discussions with participants due to the risk of shame and/or stigma. However, evaluation questions were incorporated into the surveys and clinical consultations.

##### Doing (+)

The pilot study commenced in May 2023 at ZAS Augustinus and ZAS Vincentius. From December 2023, patients from UZA were also enrolled. After a couple of months, posters about the project were displayed in the emergency rooms in the ZAS hospitals to remind emergency physicians of inclusions. Additionally, information about the project was disseminated among emergency nurses at the ZAS hospitals and UZA to remind them to inform emergency doctors about eligible patients.

##### Reflecting and evaluating implementation (+)

Meetings with deliverers and stakeholders were organised to evaluate the implementation process. Additionally, the important steps of the implementation process were discussed during clinical meetings. Finally, the implementation was also continuously discussed and evaluated during monthly research meetings.

##### Reflecting and evaluating innovation (+)

The effect of the pilot study was evaluated, as discussed further in this article. Also, the opinions and experiences of participants and their parents were evaluated. In the organised meetings with deliverers and researchers, the innovation itself was also evaluated.

##### Adapting (+)

No major changes were necessary during the implementation process, as the innovation was based on an established model in the Netherlands. However, some adaptations were made, based on experiences from researchers and/or stakeholders during implementation. First, the emergency physicians were asked to check and provide the contact details of the participants when it appeared by the medical doctors and child psychologist they were often missing from the EHR. Second, follow-up consultations were sometimes performed by telephone instead of in person, when requested by participants and their parents. Halfway through, the parent survey was divided into two separate surveys to ensure that the psychological questionnaires were completed by both parents and/or guardians. This was advised by both the child psychologist and some parents. Finally, during the running time of the intervention, it was decided in consultation with the emergency physicians, to also include patients with AAI who left the hospital under the supervision of a parent instead of staying overnight.

#### Outcome addendum

##### Adoptability (±)

Cited by the paediatricians of UZA, 16- and 17-year-olds were not included in UZA, as only paediatricians were allowed to include patients and not emergency physicians, who normally treat this age group. Also, the kick-off at UZA was delayed due to the busy schedules of the emergency room staff. Additionally, despite multiple attempts, implementation was not established at other ZAS hospitals (namely, the former ZNA hospitals), as explained by the research team. In this context, a patient with AAI who was initially admitted at a former ZNA hospital but whose mother found their way to the SPIRIT by themselves, said: *‘I found the SPIRIT on the internet because I was actively searching for help. I would also advise offering information about follow-up treatment at this hospital’ [Parent 3].*

##### Implementability (±)

During the intervention’s implementation, emergency physicians reported seeing fewer eligible patients than expected initially. In this regard, they had the feeling there were fewer AAI admissions among adolescents than previously and more among young adults. Also, emergency physicians reported that some potentially eligible patients did not fit the inclusion criteria. These were AAI patients who would need an overnight admission at the emergency room, but left the hospital under the supervision of their parents. To be able to offer SPIRIT also to these patients, the inclusion criteria were changed during implementation, so that AAI patients who were relatively soon discharged under the supervision of a parent could still be included. Some emergency physicians also gave the feedback that inclusion in the study was not a priority when there were severe psychiatric problems. Emergency physicians at the ZAS hospitals also reported that most eligible 16- and 17-year-old patients refused. In UZA, the head paediatrician reported that the paediatricians potentially missed cases during the weekend when there was not always a paediatrician working. During the evaluation sessions with the research team and emergency physicians, it also came forward that not all emergency physicians were familiar with the intervention 6 months after kick-off and/or did not see the intervention as necessary.

##### Sustainability (-)

During the implementation of the intervention, ZAS hospitals received federal financing for a psychological outpatient clinic for alcohol intoxication in young people (pilot project). Therefore, the hospital decided to continue the intervention after finishing the SPIRIT pilot study; however, without the involvement of paediatricians, as no funding for paediatricians was included in this federal financing. The SPIRIT had to be discontinued after the pilot study due to the lack of funding from the federal government, as well as the completion of the research funding for the University of Antwerp research chair ‘Reinier de Graaf Youth and Alcohol’.

### Preliminary effect analysis

#### Patient flow

Five of the 27 eligible patients (based on BAC screening) at the ZAS hospitals were included in the study. This corresponds to a reach of 18.5% (5/27). In three of the 27 patients, the study was discussed, but participation was declined by parents and/or the patient. Three of the 27 eligible patients were excluded because they were not hospitalised overnight. This was before the inclusion criteria changed, and patients admitted but not hospitalised at the ED were also considered eligible for inclusion. In the remaining 21 eligible patients, SPIRIT was not mentioned in the EHR. In addition, three patients who did not undergo BAC screening were included and were therefore not considered in the measurement of the reach of the intervention. All 5 included ZAS patients were referred from the ZAS Augustinus and none from the ZAS Vincentius.

At UZA, 16 eligible patients were screened based on a positive BAC during the study’s duration. Of them, two patients were included, and two patients declined participation. This corresponds to a reach of 12.5% (2/16). However, no study protocol existed for 16- to 17-year-old patients at UZA. The reach among 10- to 15-year-olds was 40.0%. In the other 12 eligible patients, SPIRIT was not discussed or mentioned in the EHR. Additionally, one 18-year-old UZA patient was included at the patient’s and parents’ request despite his age exceeding the inclusion criteria.

**Fig. 3:**
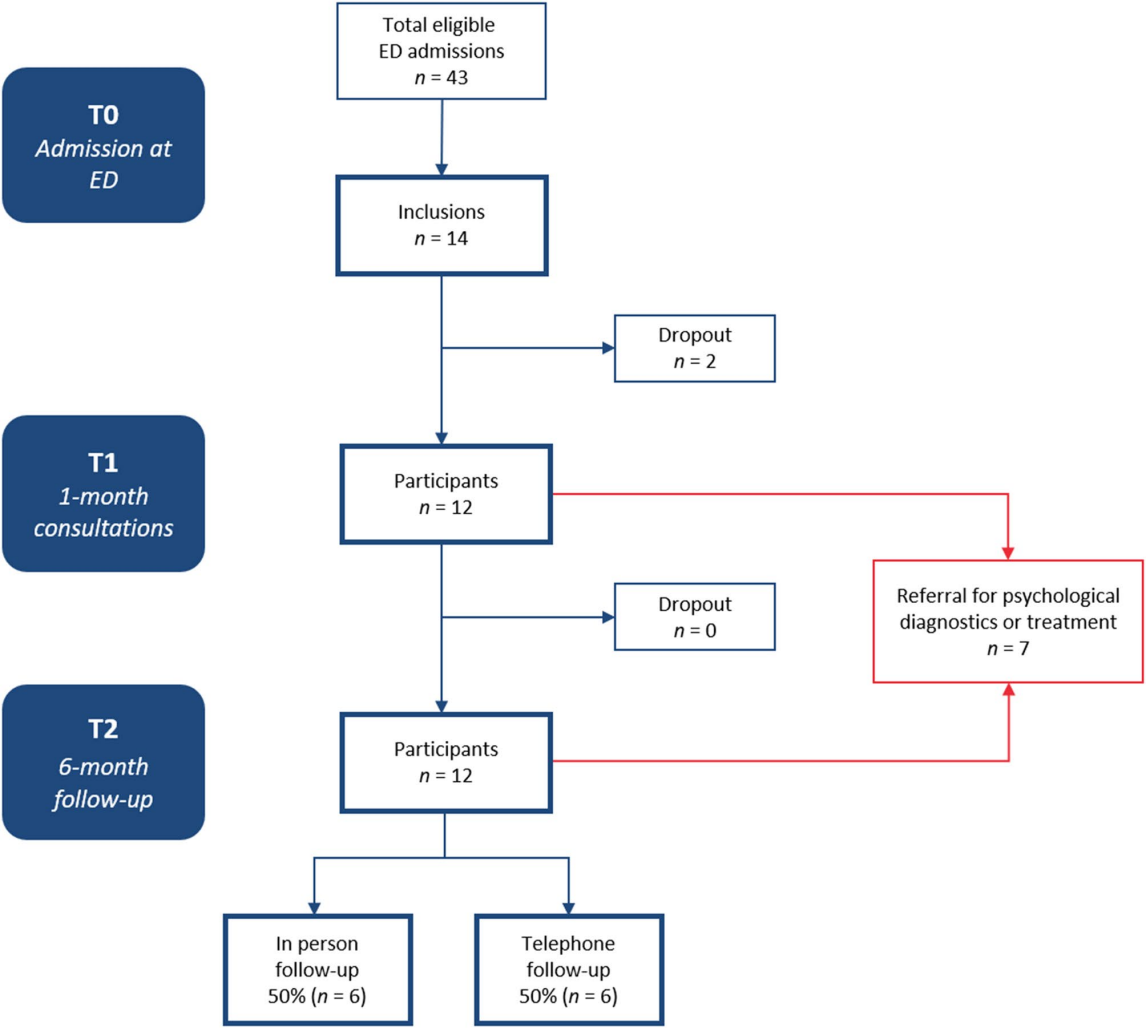
Participant flow diagram of the SPIRIT pilot study

Three patients were included after parents reached out to SPIRIT themselves. Two were initially admitted at one of the ZAS hospitals not included in the study, and one Antwerp patient was initially hospitalised in the Netherlands. Thus, in total, 14 patients were included in the study, of whom 2 dropped out after being included at the ED (T0) before completing the baseline questionnaires. The remaining 12 patients underwent the complete intervention. The flow of participants is also illustrated in Fig. 3.

#### Participant characteristics

The baseline characteristics of the participants are presented in Table 3. The mean age of the 14 included patients was 15.4 years (SD 1.6), the median age was 15.0 [IQR 14.0–17.0], and 50.0% were male. Their mean BAC at the time of admission was 2.24 g/L, corresponding with approximately 7 to 11 standard glasses of alcohol. A urine drug screening was performed on 4 participants (28.6%). In all of these cases, the drug screening was negative. However, one participant who did not undergo a urine drug screening reported the use of cannabis. Most participants (85.7%) were transferred to the hospital by ambulance. Their median GCS was 14 [IQR 12–14], with 90.1% of the participants having a GCS of 14 or lower, meaning a reduced level of consciousness. Additionally, one participant had experienced a previous AAI admission (7.1%), and seven participants had experienced drunkenness previously (50.5%). Two participants (14.3%) were diagnosed with a psychological disorder, namely ADHD, and four (28.6%) were engaged in treatment with a psychologist or psychiatrist. Of the 12 participants in the follow-up trajectory, the median CRAFFT 2.0 score was 3.0 [IQR 3.0–4.0], and 91.7% had a score of 2 or higher, indicating a high likelihood of a substance use disorder according to the DSM-5 criteria [47].

**Table 3:** Baseline characteristics of patients enrolled in the SPIRIT pilot study

Characteristic	Total population
	*n* = 14
**Age (years), n = 14**	
Mean (SD)	15.4 (1.6)
Range	12–18
**Sex, n(%), n = 14**	
Male	7 (50.0)
**Blood Alcohol Concentration (g/L), n = 11**	
Mean (SD)	2.24 (0.75)
Range	1.20–3.71
**Urine drug screening, n(%)**	
Performed	4 (28.6)
Positive screening	0 (0.0)
**Glasgow Coma Scale n(%), n = 11**	
Reduced level of consciousness (≤14 points)	10 (90.9)
Normal level of consciousness (15 points)	1 (9.1)
**Transport to ED, n(%), n = 14**	
By family	2 (14.3)
By ambulance	8 (57.1)
By ambulance specialised care (MUG)	4 (28.6)
**Previous alcohol incidents, n(%)**	
Previous AAI	1 (8.3)
Previous drunkenness	7 (58.3)
Previous accident due to alcohol	1 (8.3)
**Treatment, n(%), n = 14**	
Psychologist	3 (21.4)
Psychiatrist	1 (7.1)
**Psychological diagnosis, n(%), n = 14**	
ADHD	2 (14.3)
**Current education, n(%), n = 12**	
Primary education	1 (8.3)
Vocational secondary education	1 (8.3)
Regular secondary education	10 (83.3)
**Living situation, n(%), n = 12**	
Traditional family	4 (33.3)
Single-parent and/or reconstituted family(ies)	8 (66.7)
**Family position, n(%), n = 12**	
Eldest child	2 (16.7)
Middle child	2 (16.7)
Youngest child	7 (58.3)
Only child	1 (8.3)

#### Outcomes

**Fig. 4:**
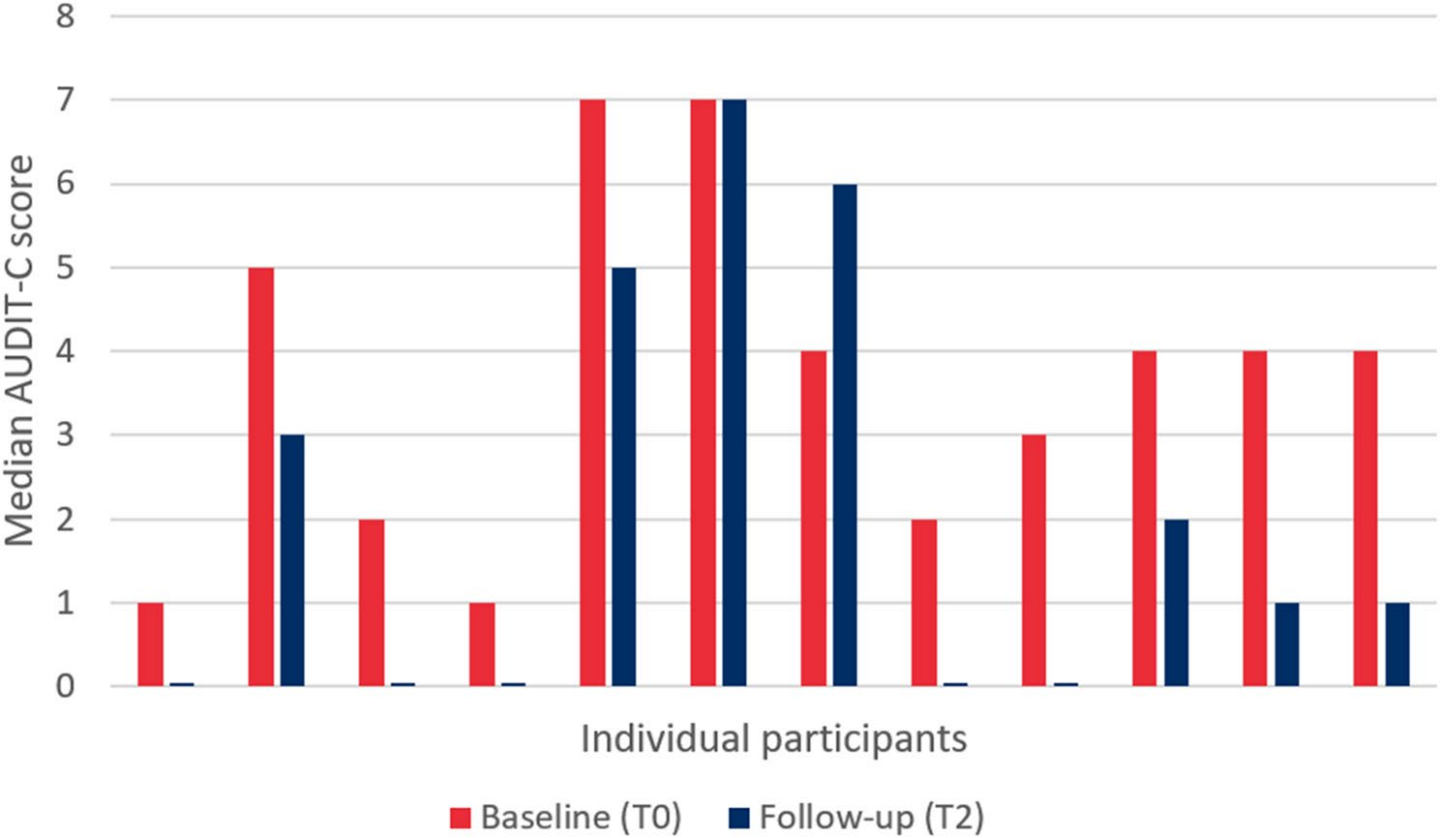
Median AUDIT-C scores (measurement of problematic alcohol use) of individual participants of the SPIRIT at baseline and follow-up

Primary and secondary outcomes, including the p-value of statistical tests, are presented in Table 3 and explained in detail below. Participants at follow-up had a statistically lower median AUDIT-C score than at baseline (*p* = 0.013). The median AUDIT-C score of participants was 4.0 [IQR 2.0–4.8] at baseline and 1.0 [IQR 0.0–4.5] at follow-up. The individual median AUDIT-C scores of participants at baseline and follow-up are presented in Fig. 4. As shown in this diagram, the AUDIT-C scores of almost all individuals decreased during the intervention period. However, the score remained the same in one patient and increased in another patient.

**Table 4:** Primary and secondary outcome variables of patients enrolled in the SPIRIT pilot study

Characteristic	Baseline (T0)	Follow-up (T2)	p-value
**AUDIT-C, n = 12**			
Median [IQR]	4.0 [2.0–4.8]	1.0 [0.0–4.5]	**0.013**
Above cutoff score (≥3), n(%)	8 (66.7)	4 (33.3)	0.125
**Binge drinking, n(%), n = 11/12**			
Never	2 (18.2)	7 (58.3)	0.125
Sometimes (less than monthly - weekly)	9 (81.8)	5 (41.7)	
**Child Behaviour Checklist, n = 12**			
(Sub)clinical score on CBCL or YSR	9 (75.0)	7 (58.3)	0.500
**Rule setting mother alcohol < 18y.o., n = 12**			0.139
Never	7 (58.3)	5 (41.7)	
Only on special occasions	4 (33.3)	2 (16.7)	
Only > 16y.o.	1 (8.3)	5 (41.7)	
Always	0 (0.0)	0 (0.0)	
**Rule setting father alcohol < 18y.o., n = 6/7**			0.785
Never	2 (33.3)	2 (28.6)	
Only on special occasions	2 (33.3)	3 (42.9)	
Only > 16y.o.	1 (16.7)	1 (14.3)	
Always	1 (16.7)	1 (14.3)	
**CBQ, median [IQR]**			
Adolescent - Mother (*n* = 12)	6.0 [2.0–10.8]	1.5 [1.0–5.8]	**0.044**
Adolescent - Father (*n* = 11)	5.0 [1.0–9.0]	2.0 [1.0–7.0]	0.102
Mother - Adolescent (*n* = 12)	8.5 [1.3–14.8]	3.5 [1.3–6.8]	**0.037**
Father - Adolescent (*n* = 9/7)	3.0 [1.5–12.5]	1.0 [1.0–4.0]	0.783
**Relationship rating, median [IQR]**			
Adolescent - Mother (*n* = 12/11)	6.0 [5.3–8.0]	8.0 [7.0–10.0]	**0.024**
Adolescent - Father (*n* = 11/10)	8.0 [6.0–8.0]	8.0 [7.8–8.5]	0.109
Mother - Adolescent (*n* = 12)	8.0 [6.3–9.0]	8.5 [7.3–10.0]	0.277
Father - Adolescent (*n* = 9/7)	8.0 [5.5–8.0]	8.0[8.0–9.0]	**0.046**
**Parenting style, mean (SD)**			
Mother (*n* = 12)	3.3 (1.1)	3.0 (0.8)	0.151
Father (*n* = 9/7)	3.3 (0.6)	2.7 (0.7)	**0.046**
**Smoking last year/last 6 months, n(%), n = 12**			1.00
No	5 (41.7)	8 (66.7)	
Yes, but stopped	7 (58.3)	1 (8.3)	
Yes, currently	0 (0.0)	3 (25.0)	
**Cannabis use last year/last 6 months, n(%), n = 12**			0.250
No	7 (58.3)	10 (83.3)	
Yes	5 (41.7)	2 (16.7)	
**Other illegal drug use last year/last 6 months, n(%), n = 12**			1.00
No	11 (91.2)	11 (91.2)	
Yes	1 (8.3)	1 (8.3)	

Bold indicates a statistically significant result

Regarding the secondary outcomes, 1 of the 12 participants (8.3%) experienced a recurrent hospital admission because of AAI between T0 and T1. Furthermore, the frequency of binge drinking decreased among participants. At baseline, 9 participants reported binge drinking sometimes (81.8%), but at follow-up, this changed to 5 participants sometimes (41.7%) and 7 who never (58.3%) engaged in binge drinking. However, this decline was not statistically significant (*p* = 0.125).

Additionally, a non-statistically significant decrease in internalising and/or externalising problems was observed at follow-up compared to baseline (*p* = 0.500). When examining the rule-setting behaviour of parents, no statistically significant changes were observed among both mothers (*p* = 0.139) and fathers (*p* = 0.785). However, the parent-child interaction measured with the CBQ improved over the intervention for all underlying relationships, with a statistically significant difference noted in the relationships between the adolescents and their mothers (*p* = 0.044 for adolescents and *p* = 0.037 for mothers). This is also reflected in all the subjective ratings of the relationships, which remained either stable or improved over time. Similarly, the adolescents’ rating of their relationship with their mothers improved significantly, from 6.0/10 to 8.0/10 (*p* = 0.024). Additionally, the mothers’ rating of their relationship with their children improved from 8.0/10 to 8.5/10, although the change was not statistically significant (*p* = 0.277). The parenting style (PS score) improved for both parents; however, the improvement was larger and statistically significant among fathers only (*p* = 0.046).

Next to the outcomes described above, we also measured perceived effect at follow-up. The majority of participants (9/12, 75.0%) stated that the SPIRIT made them drink less alcohol. For 2 participants (16.7%), this was not the case, and 1 participant (8.3%) did not have an opinion about this statement. Additionally, 6 of the 12 participants (50.0%) agreed with the statement that their relationship with their parent(s) improved due to the SPIRIT. Four participants (33.3%) disagreed with this statement, and 2 participants (16.7%) did not have an opinion about it. Among parents, 9/19 (47.4%) agreed that the intervention improved the relationship with their child, 6 disagreed (31.6%), and 4 (21.1%) did not have an opinion.

Furthermore, no statistically significant differences in smoking, cannabis use and other illegal drug use were seen between baseline and follow-up (*p* = 1.00, *p* = 0.250, and *p* = 1.00, respectively).

Of the 12 participants, 7 were referred for further diagnostics or treatment, including psychologist services, pre-addiction treatment, and/or family therapy. One of those seven participants also received an additional consultation with the SPIRIT psychologist after the intervention’s finalisation. Additionally, in one participant, a new treatment was started during the intervention time, namely a hospitalisation in a mental health institute. Furthermore, three participants received a new psychological diagnosis, namely depression (two times) and ADHD (one time). This means the number of participants with a diagnosed psychological disorder increased from 2 (14.3%) to 5 (35.7%).

## Discussion

The SPIRIT pilot study aimed to gain a deeper understanding of the barriers and facilitators to implementing a follow-up treatment program for adolescents with AAI in Antwerp, Belgium, by utilising the CFIR framework. Furthermore, a preliminary evaluation of the effect of SPIRIT was assessed to measure the potential benefits of the pilot intervention for participants and their parents.

The qualitative analysis of the pilot study revealed that most CFIR constructs served as facilitators for implementing the SPIRIT. We counted 21 facilitators out of the 38 included constructs, mainly under the domains ‘innovation’ and ‘implementation process’. In total, 15 constructs were a combination of a facilitator and barrier, mainly under the domains ‘outer setting’, ‘inner setting’ and ‘individuals’. Only two constructs were considered primarily as barriers: financing (outer setting) and sustainability (outcome addendum). Therefore, we conclude that improvements could be made mainly in the domains of outer setting and inner setting, so that the long-term finance will be better covered and sustainability will be prioritised. Additionally, the quantitative results of the quasi-experimental effect analysis showed a statistically significant decrease in the median AUDIT-C score, indicating that participants had a significantly lower risk of alcohol misuse at follow-up compared to baseline, although the sample size was small. We hypothesise that the psycho-education and motivational interviewing components of the intervention contributed to this decreased problematic alcohol use, in line with previous research [17–19]. However, causal associations between the intervention and problematic alcohol use are difficult to make, as the intervention lacked randomisation and a control group.

Moreover, the relationship between participants and their parents improved at follow-up. More specifically, a statistically significant improvement was seen in the conflict behaviour between participants and their mothers. A hypothesis here could be that due to the thorough consultations with the psychologist and medical doctors, the participants gained a better understanding of their parents’ concerns regarding their alcohol-drinking behaviour, positively influencing the underlying relationship. Literature shows that fathers often experience more barriers in seeking mental help compared to mothers [48], which is reflected, for instance, by the more challenging recruitment of fathers in parenting programs [49]. Consequently, parenting concerns and subsequent strategies are often primarily represented by the mother, which could explain the improved relationship adolescents experience with their mothers. This is probably strengthened by the fact that fathers were explicitly invited in the SPIRIT, making them more involved in the parenting situation, which potentially contributed to participants experiencing conflict with both their parents, resulting in fewer conflict situations with their mother only. Secondly, a statistically significant improvement was seen in how fathers experience the relationship with their children and in their parenting style. The consultations during the intervention and the increased talking between participants and their parents could have led to a better parental understanding of the context in which the alcohol intoxication occurred. It is possible that this increased understanding in the context of the child led to improved parenting skills and, at the same time, an improved parent-child relationship from the father’s perspective. Surprisingly, parents did not apply stricter rules regarding the alcohol drinking of their children after the intervention, in contrast to similar programs [22]. Parents did state the intervention made them talk more about alcohol with their children. This, together with a potentially increased internal motivation to drink less alcohol among their children, might have led to an increased trust in their children and, therefore, a reduced need to apply stricter rules. Another explanation could be that the existing parental rules did not need to become more stringent due to better monitoring and compliance with the already existing rules. Finally, the preliminary analyses revealed that nine participants had a (sub)clinical score on the YSR and/or CBCL scale for internalising, externalising or total problems at baseline, indicative of undiagnosed psychological disorders. In this regard, seven of the twelve participants were referred for additional screening and/or treatment, of which three received a new psychological diagnosis. Therefore, it is believed that the intervention supported the screening for underlying psychological disorders, which is crucial to prevent future alcohol misuse among these patients [14]. In conclusion, these preliminary findings support the hypothesis that a follow-up program for adolescents with AAI may be beneficial for both participants and their parents, which aligns with previous research [22, 27].

The relevance of the SPIRIT was demonstrated not only by the decrease in alcohol misuse among participants and the low dropout rate, but it was also emphasised in the qualitative analyses. This was evident in the fact that no treatment protocol for adolescents with AAI was in use at the time of implementation. Additionally, the experiences and opinions of participants and their parents regarding the initial AAI admission highlighted the need for the intervention, as most participants reported that nothing changed at home after the admission, and that they needed a better follow-up after the ED admission. On the contrary, the perceived effect of the intervention was positive, with most participants and parents stating that the SPIRIT made them drink less and a perceived improvement in the parent-child connection.

The implementation process evaluation also provided insight into how the SPIRIT could be improved to achieve a higher impact. Firstly, it is expected that the potential effect of the intervention may increase if prevention is initiated more extensively at the time of ED admission or, more precisely, at the moment of patient awakening. This could be done by setting up a consultation with an expert at discharge, as some parents recommended. In this way, it is possible to make use of the ‘teachable moment’ of the hospital admission, which could prompt behavioural change when integrating it with clinical-patient interaction [50, 51]. However, a challenge arises here, as tasks for healthcare professionals at EDs should be as little time-consuming as possible, as shown by our process evaluation. Nevertheless, also without this clinical-patient interaction directly after awakening of the patient, the SPIRIT already showed promising results in this pilot study. Secondly, the evaluation of the implementation process could provide direction on how to achieve a larger sample size in similar future research. Here, the flow of patients might have been the biggest challenge. For instance, although the ZAS Augustinus and ZAS Vincentius participated in the intervention, no patients were included at the ED of ZAS Vincentius. Moreover, despite multiple efforts, other ZAS hospitals were not participating in the intervention. Also, the 16- and 17-year-olds from UZA were not referred to the intervention. This likely resulted in a low reach of the intervention. We might, therefore, conclude that the flow of patients works most conveniently in the hospital where the follow-up program is in place. However, other reasons might have played a role in the small reach, such as hesitancy to participate by patients and/or their parents or limited time for the emergency physicians to organise the inclusions, although deficient information on this was available from the evaluation of the implementation process.

The findings of the SPIRIT pilot study should be interpreted with consideration of some limitations in the study design. Firstly, the pilot study included a small sample size of 14 participants, of whom 12 completed the intervention. This limited number influenced the power of the analyses, making it more difficult to detect small changes in outcomes and to extrapolate the results to the overall population. In other words, this small sample size might undermine the internal and external validity of the findings [52], and therefore, the results should be interpreted cautiously. Secondly, as the SPIRIT was a complex, newly developed intervention, a pilot study without a control group was considered an appropriate study design at the start [53]. However, the absence of a control group generated a risk of selection bias. Here, the possibility exists that specific groups of patients chose to participate in the intervention, for instance patients who were motivated to change. Unfortunately, we did not obtain additional information on why patients declined participation. To get insight into the number of eligible patients admitted with AAI during the running time of the intervention, we made an estimation of missed cases based on BAC screening retrospectively, revealing a total of 36 missed cases and a reach of 18.5% and 12.5% in the two hospitals, respectively. However, this reach is possibly even an underestimation, as previous research shows only 83% of adolescents presenting with AAI at EDs undergo BAC screening [4]. Finally, the specific context of the city of Antwerp (Belgium) is another limitation, as it may not be representative of implementation in different regions or countries. Different barriers (and facilitators) are likely to be encountered when implementing a similar intervention in a different context, as the specific healthcare system and stakeholders have a significant influence. Therefore, the results of the implementation process evaluation of this pilot study cannot be extrapolated to other contexts without caution.

Despite these cautionary notes, we encourage further studies with larger sample sizes to determine if these promising findings can be confirmed. A randomised controlled trial could be especially interesting as it would decrease the chance of selection bias by introducing a control group and allow for a better internal validity. We recommend that future research focus on increasing the sample size by improving the flow of eligible patients. This could potentially be reached by ensuring the full commitment of participating hospitals and stakeholders and involving the medical management of participating hospitals beforehand. In this respect, agreements between hospitals regarding the mutual referral of patients could be helpful. Additionally, external financing could be crucial in scaling up and embedding the intervention in the healthcare system. Finally, it would be interesting to involve participants and their parents in the design of the intervention. Although this was difficult at the beginning of the SPIRIT development, since no similar intervention was in place in Belgium at that time, participants who have completed the intervention may now be available to contribute to the design of parts of the intervention.

The findings of the SPIRIT pilot study show the need for a multidisciplinary follow-up treatment for adolescents with acute alcohol intoxication in Belgium. Both facilitators and barriers to implementation have been identified, revealing a strong innovation design (a high adaptability and triability, for instance) and implementation process (a thorough context assessment and the possibility to tailor strategies, for instance) and areas for improvement in both the outer and inner setting domains (for instance, patient flow and financing). Additionally, the effect evaluation of the SPIRIT shows promising results, both on the alcohol misuse among the participants and on the parent-child interaction and parenting skills. The intervention was also perceived positively by both participants and their parents. To conclude, the insights of this mixed-method pilot study could facilitate the implementation of a similar intervention in other Belgian hospitals. With additional funding, the intervention in the SPIRIT study could be further tested on a larger scale with a control group and adapted based on the lessons learned to reach more patients and potentially develop into a national secondary prevention program.

## Conclusions

This SPIRIT pilot study aimed to qualitatively assess the implementation process of SPIRIT, a 6-month multidisciplinary outpatient follow-up program for adolescents who experienced an admission due to alcohol intoxication in Antwerp (Belgium), and to quantitatively analyse the interventions’ effect. The findings show the need for the intervention, as no protocol for adolescents with AAI and their parents was in place before, and both participants and their parents stated the admission itself did not lead to changes at home. The perceived effect of the intervention was positive, with a decrease in alcohol use, and an increased connection between the child and their parents. The low sample size of the pilot study might be explained by difficulties faced in the patient flow between participating hospitals. Therefore, ensuring the full commitment of participating hospitals and important stakeholders from the field should be considered an important focus point in future research. Despite this low sample size, the preliminary effect analysis of this study shows promising results in decreasing problematic alcohol use, improving parent-child interaction and screening for psychological disorders. However, these promising results should be further tested in studies with larger sample sizes and included control groups. The barriers (mainly in the inner and outer setting domains, for instance patient flow and financing) and facilitators (mainly in the innovation and implementation process domain, for instance triability, context assessment and tailoring strategies) assessed in this study using the CFIR framework, could be considered by researchers, physicians and policy makers when implementing similar follow-up programs in healthcare contexts.

## Electronic supplementary material

Below is the link to the electronic supplementary material.

Supplementary Material 1

## Data Availability

The datasets used and/or analysed during the current study are available from the corresponding author upon reasonable request.

## Abbreviations

AAI: Acute alcohol intoxication
MI: Motivational interviewing
ED: Emergency department
SPIRIT: Screening and Personalised Feedback Intervention for Alcohol-Intoxicated Teenagers
ZAS: ‘Ziekenhuis aan de Stroom’, Antwerp-based hospitals
UZA: Antwerp University Hospital
CFIR: Consolidated Framework for Implementation Research
PAR: Participatory Action Research
BAC: Blood alcohol concentration
GCS: Glasgow Coma Scale
AUDIT: Alcohol Use Disorders Identification Test
VAD: Expertise Centre for Alcohol and other Drugs
CBCL: Child Behaviour Checklist
YSR: Youth Self Report
CBQ: Conflict Behaviour Questionnaire
PS: Parenting Scale
HER: Electronic health record
CRAFFT questionnaire: ‘Car, Relax, Alone, Forget, Friends, Trouble’ questionnaire
SD: Standard deviation
IQR: Interquartile range
