# Supplementary material for: Implementation process evaluation and preliminary effect analysis of an outpatient multidisciplinary follow-up program for adolescents with acute alcohol intoxication in Belgium: the SPIRIT pilot study
Source: Addict Sci Clin Pract. 2025 Dec 11;20:96. doi: 10.1186/s13722-025-00629-z (PMC12720440; doi:10.1186/s13722-025-00629-z)
Supplement: Supplementary file 1 — Supplementary Material 1 [file 13722_2025_629_MOESM1_ESM.docx]

# English translation of the Dutch semi-structured interview with Head of Paediatrics

Name:
Date:

1. Introduction
2. Is alcohol intoxication among minors considered a problem in your hospital? How is it perceived by you personally and by your colleagues?
3. What is your estimate of the annual number of admissions of minors due to alcohol intoxication in your hospital?
4. How would you assess the severity of these admissions? For instance, in terms of clinical presentation, required interventions, or outcomes?
5. Have you observed any trends regarding the annual number or severity of admissions for alcohol intoxication among minors in recent years?
6. Could you describe the workflow for admissions of minors with alcohol intoxication in your hospital?
7. Which physicians or departments are typically involved?
8. Is the pediatrician involved even if the patient is only treated in the emergency department?
9. Is there a standard treatment plan or protocol for this patient group?
10. Are there specific guidelines or agreements for the treatment these cases?
    For example: when to perform a blood alcohol concentration test, urine toxicology, electrolyte analysis, blood gas, temperature and glucose checks, ECG, or IV placement; criteria for admission; involvement of parents; provision of education or counseling; follow-up by a pediatrician.
11. Are child psychologists, pedagogical staff, or other disciplines involved in the management of these cases?
12. What treatment options—both inpatient and outpatient—are available for this patient group, and how often are they used?
13. What is your opinion on establishing a registration system for this patient group? The aim would be to gain better insight into the number and characteristics of such cases across the Antwerp region or even Flanders/Belgium. How do you envision such a system working?
14. Our idea is to set up and implement an outpatient clinic for minors who experienced a hospital admission due to alcohol intoxication, in the form of pilot project. The clinic could offer patients follow-up care for several months, by consultations with a paediatrician and a child psychologist, including education and the involvement of parents. The goal would be to prevent recurrence.
    What are your thoughts on such an outpatient clinic?
15. Closing
